# Vertical Orientation of Liquid Crystal on Polystyrene Substituted with *n*-Alkylbenzoate-*p*-oxymethyl Pendant Group as a Liquid Crystal Precursor

**DOI:** 10.3390/polym13132058

**Published:** 2021-06-23

**Authors:** Kyutae Seo, Hyo Kang

**Affiliations:** BK-21 Four Graduate Program, Department of Chemical Engineering, Dong-A University, 37 Nakdong-Daero 550 Beon-gil, Saha-gu, Busan 49315, Korea; kyutae@donga.ac.kr

**Keywords:** anisotropic material, liquid crystal, orientation layer, polystyrene, *n*-alkyl-*p*-hydroxybenzoate

## Abstract

We synthesized a series of polystyrene derivatives modified with precursors of liquid crystal (LC) molecules via polymer modification reactions. Thereafter, the orientation of the LC molecules on the polymer films, which possess part of the corresponding LC molecular structure, was investigated systematically. The precursors and the corresponding derivatives used in this study include ethyl-*p*-hydroxybenzoate (homopolymer P2BO and copolymer P2BO#, where # indicates the molar fraction of ethylbenzoate-*p*-oxymethyl in the side chain (# = 20, 40, 60, and 80)), *n*-butyl-*p*-hydroxybenzoate (P4BO), *n*-hexyl-*p*-hydroxybenzoate (P6BO), and *n*-octyl-*p*-hydroxybenzoate (P8BO). A stable and uniform vertical orientation of LC molecules was observed in LC cells fabricated with P2BO#, with 40 mol% or more ethylbenzoate-*p*-oxymethyl side groups. In addition, the LC molecules were oriented vertically in LC cells fabricated with homopolymers of P2BO, P4BO, P6BO, and P8BO. The water contact angle on the polymer films can be associated with the vertical orientation of the LC molecules in the LC cells fabricated with the polymer films. For example, vertical LC orientation was observed when the water contact angle of the polymer films was greater than ~86°. Good orientation stability was observed at 150 °C and with 20 J/cm^2^ of UV irradiation for LC cells fabricated with the P2BO film.

## 1. Introduction

Liquid crystals (LCs) are attractive materials, which have intermediate phase between ordinary liquids and three-dimensional solids. The LC molecules, which are anisotropic materials, play an imperative role in materials science when it comes to investigate the correlation between chemical structure and physicochemical properties. In addition, the LC molecules can be used in numerous applications owing to their extraordinary physicochemical properties, such as dielectric anisotropy, optical anisotropy, and susceptibility to external electric field and/or magnetic fields [1]. It is important to orient the LC molecules in one direction, which plays an imperative role in the industrial applications as well as scientific research [2,3,4,5,6,7,8,9]. The most well-known example of these efforts is the development of the liquid crystal display (LCD), which involves an orientation layer, and is widely used in portable display devices such as smartphones [10,11]. It has been found that liquid crystal (LC) molecules can be oriented by the anisotropic characteristics of a substrate surface via numerous contact and noncontact methods, such as mechanical rubbing, stretching, lithography, polarized ultraviolet (UV) radiation, and ion beam treatment [12,13,14,15,16,17,18,19]. Due to its simplicity and rapidity, the mechanical rubbing of polymeric surfaces is the most commonly used contact method for controlling not only the orientation direction, but also the pretilt angle of the LC molecules [20,21]. Films of polyimide derivatives have been widely employed as LC orientation layers using the rubbing technique because the polymer films provide considerable stability for the LC orientation [22,23,24,25,26]. However, the rubbing process can result in unexpected problems, such as dust generation, physical damage, and electrostatic charge on the orientation layer surfaces [27,28,29]. Thus, noncontact methods to orientate the LC molecules have been investigated to overcome the drawbacks of the rubbing method. Photoalignment has been proposed as a promising noncontact orientation technology for next-generation LC display applications, such as flexible displays, because it has the advantages of cleanliness, lack of restrictions with respect to surface morphology, and suitability for large glass substrates. Numerous polymers, including a variety of photoreactive functional groups that can undergo photoisomerization, photodimerization, and photodegradation, have been studied as photoalignment layers [30,31,32]. The orientation of the LC molecules between two substrates can be classified into two groups according to the degree of variation from the LC director in the cells. For example, LC molecules in planar, tilted, and vertically oriented LCs preferably align in one direction; however, molecules in splay, twist, bend, and hybrid LCs are not aligned in one direction [33]. Conventional display panel technologies, such as the twisted nematic mode, which have been generally used for LCDs, have the disadvantages of a narrow viewing angle and low contrast ratio [34,35]. Therefore, other modes with wide viewing angles and high contrast ratios, including the in-plane switching (IPS) mode, fringe-field switching (FFS) mode, and vertical alignment (VA) modes have been developed [36,37,38]. Among these modes, the VA mode has an especially high contrast ratio owing to minimal retardation [39,40,41]. Additionally, nematic LCs, wherein the director is oriented vertically to the surface of the substrate, have been studied for sensor applications because of their susceptibility to small perturbations and any binding events [42,43,44,45], which can be observed not only with the naked eye but also with optical techniques such as polarized optical microscopy (POM) [46,47]. The vertical orientation of LC molecules can be determined using the relationship between the surface tension of the liquid crystalline material (γ_lc_) and the surface energy of the orientation layer (γ_s_) according to the Friedel–Creagh–Kmetz experimental rule [48,49,50]. For example, liquid crystalline materials can be oriented vertically on the substrate surface when γ_lc_ > γ_s_. Polyimides (PIs) with side chains, such as long alkyl groups, have been studied in order to achieve the vertical orientation of LC molecules. However, the post-baking and cleaning temperatures required to prepare the orientation layer from PI derivatives are too high for the manufacture of flexible plastic products [51,52,53]. Recently, vertically oriented LC layers using polystyrene (PS) derivatives synthesized via a polymer substitution reaction have been developed for electro-optical applications, including flexible displays, owing to their advantages of low temperature processability and superior optical transparency. PS derivatives, grafted with natural extracts, long alkyl groups, or fluoroalkyl groups, have been developed to orient LC molecules vertically on substrates using noncontact methods. For example, the vertical orientation of LC molecules in LC cells fabricated with PS derivatives substituted with natural extracts, such as capsaicin, eugenol, and vanillin, was observed when the substituent ratio was greater than 60 mol%. This is due to the long alkyl groups of the natural extracts, which are related to the low surface energy owing to the steric effect of the alkyl groups on the polymer film surface [54,55,56,57].

In this study, we synthesized a series of PS derivatives with *n*-alkylbenzoate-*p*-oxymethyl side groups in order to systematically investigate the LC behavior of the orientation layer fabricated with PS derivatives structurally similar to LC molecules. The LC molecules were vertically oriented in the LC cells fabricated with these polymer films when the substituent ratio was only 40 mol%. These results indicate that similarities between the chemical structure of the orientation layer and the LC molecules are favorable for the vertical orientation of LC molecules. The synthesis and characterization of these polymers as well as the optical properties of the assembled LC cells with unrubbed polymer films were studied.

## 2. Materials and Methods

### 2.1. Materials

4-Chloromethylstyrene, 4′-pentyl-4-biphenylcarbonitrile (5CB, *n_e_* = 1.7360, *n_o_* = 1.5442, and Δ*ε* = 14.5, where *n_e_*, *n_o_*, and Δ*ε* represent the extraordinary refractive index, ordinary refractive index, and dielectric anisotropy, respectively) and silica gel were purchased from Merck Co. (Seoul, Korea). Benzophenone, sodium, and hexane were purchased from Aldrich Co. (Seoul, Korea). Ethyl-*p*-hydroxybenzoate, *n*-butyl-*p*-hydroxybenzoate, *n*-hexyl-*p*-hydroxybenzoate, and *n*-octyl-*p*-hydroxybenzoate were obtained from the Tokyo Chemical Industry Co. (Tokyo, Japan). Potassium carbonate, 2,2′-azobisisobutyronitrile (AIBN), tetrahydrofuran (THF), molecular sieves (4 Å), and *N*,*N′*-dimethylacetamide (DMAc) were acquired from Daejung Chemicals & Metals Co. (Siheung, Korea). Methanol was supplied by SK Chemical Co. (Ulsan, Korea). DMAc and ethanol were dried over molecular sieves. THF was dried by refluxing with benzophenone and sodium, followed by distillation. 4-Chloromethylstyrene was purified by column chromatography with silica gel using hexane as an eluent to remove any impurities and inhibitors (*tert*-butylcatechol and nitroparaffin). AIBN was purified by crystallization using methanol. Poly(4-chloromethylstyrene) (PCMS) was synthesized through conventional free radical polymerization of 4-chloromethylstyrene using AIBN under a nitrogen atmosphere. The mixture was cooled to room temperature and poured into methanol to obtain a white precipitate. The precipitate was further purified by Soxhlet extraction using boiling methanol to remove the remaining monomer (4-chloromethylstyrene) and low-molecular-weight PCMS. AIBN was used as an initiator. All other reagents and solvents were used as received.

^1^H NMR of PCMS (400 MHz, CDCl_3_, *δ*/ppm): *δ* = 1.01–1.88 (–*CH_2_*–*CH*–Ph–, 3H), *δ* = 4.13–4.77 (–Ph–*CH_2_*–Cl, 2H), *δ* = 6.00–7.22 (CH_2_–CH–*PhH*–CH_2_–, 4H).

### 2.2. Preparation of n-alkylbenzoate-p-oxymethyl Modified Polystyrene

The following procedure was used to synthesize all *n*-alkylbenzoate-*p*-oxymethyl-substituted polystyrenes (PABOs), where the alkyl group was –COO–(CH_2_)_n_H (2, 4, 6, and 8). The synthesis of ethylbenzoate-*p*-oxymethyl-substituted polystyrene (P2BO) is provided as an example. A mixture of PCMS (0.300 g, 1.97 mmol), ethyl-*p*-hydroxybenzoate (0.492 g, 2.96 mmol, 150 mol% compared with PCMS), and potassium carbonate (0.491 g, 3.55 mmol, 120 mol% compared with ethyl-*p*-hydroxybenzoate used substituent) in DMAc (50 mL) was heated to 70 °C and magnetically stirred at 200 rpm under a nitrogen atmosphere. The substitution reaction lasted for 24 h. Thereafter, the solution mixture was cooled to room temperature and poured into methanol to obtain a white precipitate. The precipitate was further purified by several reprecipitations from DMAc solution into methanol, and then a Soxhlet extractor was used to remove potassium carbonate and the remaining salts with boiling methanol. P2BO was obtained in >80% yield after drying under vacuum overnight.

^1^H NMR of P2BO (400 MHz, CDCl_3_, *δ*/ppm): *δ* = 0.98–2.38 (–*CH_2_–CH*–Ph–CH_2_–, –COO–CH_2_–*CH_3_*, 6H), *δ* = 4.13–4.49 (–Ph–COO–*CH_2_*–CH_3_, 2H), *δ* = 4.72–5.12 (–Ph–*CH_2_*–O–Ph–, 2H), *δ* = 6.09–8.13 (–CH_2_–CH–*PhH*–CH_2_–O–*PhH*–COO–, 8H).

Similarly, *n*-butylbenzoate-*p*-oxymethyl (P4BO, *n* = 4), *n*-hexylbenzoate-*p*-oxymethyl (P6BO, *n* = 6), and *n*-octylbenzoate-*p*-oxymethyl-substituted polystyrene (P8BO, *n* = 8) were synthesized using the same procedure, except that *n*-butyl-*p*-hydroxybenzoate (0.575 g, 2.96 mmol, 150 mol% compared with PCMS), *n*-hexyl-*p*-hydroxybenzoate (0.658 g, 2.96 mmol, 150 mol% compared with PCMS), and *n*-octyl-*p*-hydroxybenzoate (0.741 g, 2.96 mmol, 150 mol% compared with PCMS) were used instead of ethyl-*p*-hydroxybenzoate. The synthesis process of each homopolymer is described in detail in the Supplementary Materials.

^1^H NMR of P4BO (400 MHz, CDCl_3_, *δ*/ppm): *δ* = 0.86–1.81 (–*CH_2_–CH*–Ph–CH_2_–, –COO–CH_2_–*(CH_2_)_2_–CH_3_*, 10H), *δ* = 4.17–4.39 (–Ph–COO–*CH_2_*–CH_2_–, 2H), *δ* = 4.70–5.06 (–Ph–*CH_2_*–O–Ph–, 2H), *δ* = 6.09–8.11 (–CH_2_–CH–*PhH*–CH_2_–O–*PhH*–COO–, 8H).

^1^H NMR of P6BO (400 MHz, CDCl_3_, *δ*/ppm): *δ* = 0.76–1.87 (–*CH_2_–CH*–Ph–CH_2_–, –COO–CH_2_–*(CH_2_)_4_–CH_3_*, 14H), *δ* = 4.12–4.34 (–Ph–COO–*CH_2_*–CH_2_–, 2H), *δ* = 4.67–5.06 (–Ph–*CH_2_*–O–Ph–, 2H), *δ* = 5.99–8.14 (–CH_2_–CH–*PhH*–CH_2_–O–*PhH*–COO–, 8H).

^1^H NMR of P8BO (400 MHz, CDCl_3_, *δ*/ppm): *δ* = 0.77–1.78 (–*CH_2_–CH*–Ph–CH_2_–, –COO–CH_2_–*(CH_2_)_6_–CH_3_*, 18H), *δ* = 4.16–4.33 (–Ph–COO–*CH_2_*–CH_2_–, 2H), *δ* = 4.74–5.03 (–Ph–*CH_2_*–O–Ph–, 2H), *δ* = 5.95–8.18 (–CH_2_–CH–*PhH*–CH_2_–O–*PhH*–COO–, 8H).

The copolymer of P2BO, designated P2BO#, where # denotes the degree (mol%) of substitution of chloromethyl to the ethylbenzoate-*p*-oxymethyl group, was prepared using the same procedure as that for P2BO, except that <150 mol% of ethyl-*p*-hydroxybenzoate was used. P2BO20, P2BO40, P2BO60, and P2BO80 were prepared with 0.065 g (0.39 mmol), 0.131 g (0.79 mmol), 0.196 g (1.18 mmol), and 0.263 g (1.58 mmol) of ethyl-*p*-hydroxybenzoate, respectively, using excess quantities of potassium carbonate (120 mol% compared with ethyl-*p*-hydroxybenzoate). The synthesis process of each copolymer is described in detail in the Supplementary Materials.

### 2.3. Film Preparation and LC Cell Assembly

Solutions of P2BO#, PABO (P2BO, P4BO, P6BO, and P8BO) in THF (1 wt%) were filtered using a poly(tetrafluoroethylene) membrane with a pore size of 0.45 μm. Then, thin polymer films were prepared by spin-coating (2000 rpm, 60 s) onto 2.0 cm × 2.5 cm glass substrates using the 1 wt% THF solution of P2BO#, PABO (P2BO, P4BO, P6BO, and P8BO). LC cells were fabricated by assembling two polymeric layers onto two glass substrates using spacers with thicknesses of 4.25 μm. The physicochemical properties of 4-pentyl-4-cyanobiphenyl (5CB), such as surface tension, have been documented in numerous studies because of their accessible nematic temperature range (near room temperature), high positive dielectric anisotropy, and remarkable chemical stability. Therefore, 5CB was selected to fabricate the LC cells to investigate the correlation between the orientation layer and LC molecules via physicochemical interactions [58,59,60,61]. The cells were filled with nematic LCs (5CB). The manufactured LC cells were sealed using an epoxy glue.

### 2.4. Instrumentation

^1^H-nuclear magnetic resonance (NMR) spectroscopy using an MR400 DD2 (Agilent Technologies, Inc., Santa Clara, CA, USA), differential scanning calorimetry (DSC) using a Q-10 (TA Instruments, Inc., New Castle, DE, USA), and POM images of LC cells using a Nikon Eclipse E600 POL (NIKON, Inc., Tokyo, Japan) equipped with a polarizer and a Nikon Coolpix 995 digital camera (NIKON, Inc., Tokyo, Japan) were employed to characterize the synthesized materials. The static contact angles of water on the polymer films were determined using a KRÜSS DSA10 (KRÜSS Scientific Instruments Inc., Hamburg, Germany) contact angle analyzer equipped with drop shape analysis software (KRÜSS Scientific Instruments Inc., Hamburg, Germany). The contact angles for each sample were measured more than four times on three independently prepared films, and the average values were used. UV stability tests were conducted on the LC cells using a VL-6.LC lamp (λ_max_ = 365 nm, Vilber Lourmat, Paris, France) to investigate the effects of a severe environment. The exposure dose of UV irradiation on the LC cells was measured with a UV detector using a GT-513 (Giltron, Seoul, Korea).

## 3. Results and Discussion

**Figure 1:**
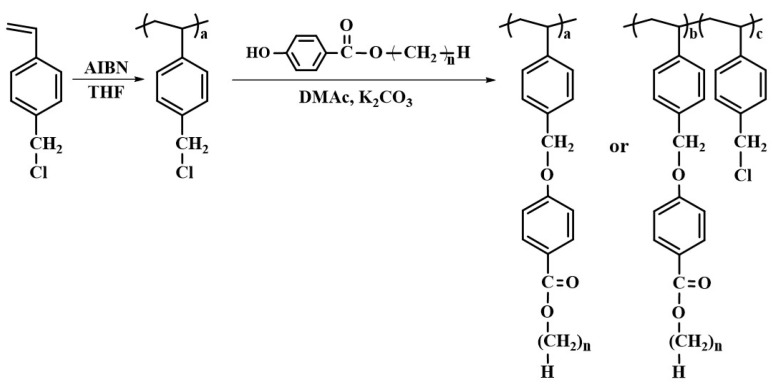
Synthetic route to ethylbenzoate-*p*-oxymethyl (P2BO# and P2BO, *n* = 2), *n*-butylbenzoate-*p*-oxymethyl (P4BO, *n* = 4), *n*-hexylbenzoate-*p*-oxymethyl (P6BO, *n* = 6), and *n*-octylbenzoate-*p*-oxymethyl-substituted polystyrene derivatives (P8BO, *n* = 8), where # represents the molar fraction of ethyl-*p*-hydroxybenzoate containing monomeric units in the polymer.

The synthetic routes used to prepare the PABO (P2BO, P4BO, P6BO, and P8BO) and P2BO# copolymers (P2BO20, P2BO40, P2BO60, and P2BO80) are shown in Figure 1. It has been reported that a conversion rate of a substitution reaction can decrease as the degree of replacement increases [62]. However, the conversion ratios in this substitution reaction of the chlorine atom in PCMS by nucleophilic compounds, such as phenolate compounds, have been observed to approximately agree with each feeding ratio of substituent according to previous studies [63,64]. The acidity of phenolic compounds can be enhanced by electron-withdrawing substituents group in ortho or para position to the hydroxyl group [65,66]. The phenol group of the *n*-alkyl-*p*-hydroxybenzoate can be readily dissociated to the proton and the phenolate anion, which is a strong nucleophile, because the structure of phenolate anion can be stabilized by resonance structures. In addition, the benzylic carbon in PCMS is relatively electron-deficient, because of the electron-withdrawing groups, namely the chlorine and phenyl groups, which are attached directly to the carbon [67]. Moreover, the structure of the transition state in this substitution reaction can be stabilized by conjugation with the benzene ring. Therefore, the high conversion rate in this polymer modification reaction can be explained not only by the electrophilicity of benzylic carbon in PCMS, but also the chemical structure stability of the phenolate anion as a nucleophile.

**Figure 2:**
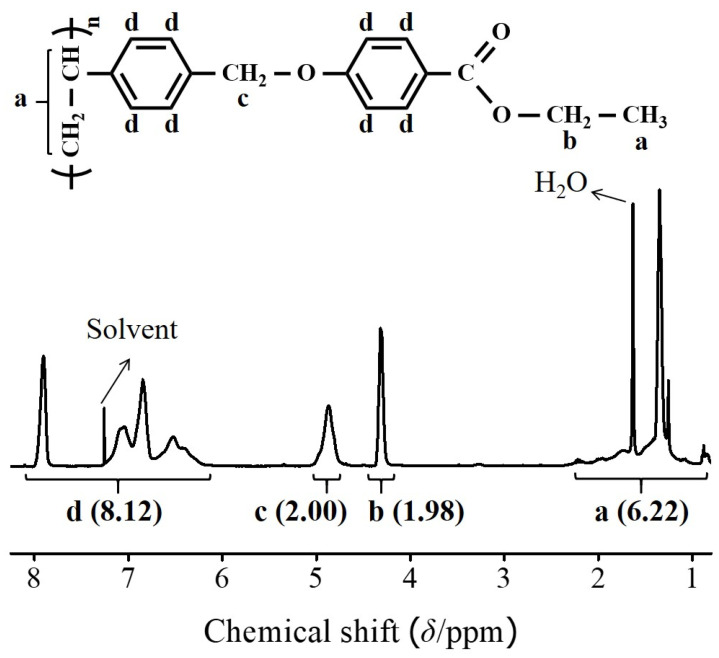
^1^H nuclear magnetic resonance (NMR) spectrum of P2BO.

Copolymers with different substitution ratios (mol%) were obtained by varying the molar ratio of ethyl-*p*-hydroxybenzoate in the reaction mixture. Almost 100% conversion of chloromethyl to oxymethyl was achieved when 150 mol% of ethyl-*p*-hydroxybenzoate, *n*-butyl-*p*-hydroxybenzoate, *n*-hexyl-*p*-hydroxybenzoate, and *n*-octyl-*p*-hydroxybenzoate were reacted with PCMS at 70 °C for 24 h. Figure 2 shows the ^1^H NMR spectra of P2BO as an example. The chemical composition of the monomeric units in the obtained polymers was confirmed using ^1^H NMR spectroscopy. The ^1^H NMR spectrum and assignment of the P2BO peaks are shown in Figure 2. The ^1^H NMR spectrum of P2BO indicates the presence of protons from the P2BO derivatives (*δ*/ppm = 0.98–2.38 (6H), 4.13–4.49 (2H), 4.72–5.12 (2H), 6.09–8.13 (8H); peaks a, b, c, and d). The degree of substitution from chloromethyl to oxymethyl was calculated to be approximately 100% by comparing the integrated area of the oxymethyl peak in the 4.72–5.12 ppm range and the phenyl group peaks in the 6.09–8.13 ppm range. The ^1^H spectra of the other polymers, including PCMS, PABO and P2BO#, are shown in Figures S1–S8. The integrations and calculations for P2BO# and PABO were performed, and the results were typically within ±10% of the expected values. Considerable challenges still exist to the exactitude of the quantification, even though quantitative NMR measurements have often been proven to be robust and reproducible. The sizes of NMR signals, which were produced from the same source, can be variable under different conditions, such as sample composition, excitation purse angle, NMR tube size, sample volume, and other experimental and geometrical parameters. The substitution ratios of each polymer calculated from ^1^H NMR spectrum should be interpreted in consideration of possibility of errors in integral values [68]. In addition, ^13^C NMR spectroscopy was used to examine the chemical structure of the polymers, respectively. Each of the spectra is shown in Figures S9–S17. These polymers were soluble in medium-polarity solvents with low boiling points, such as THF and chloroform, and in aprotic polar solvents, including *N*,*N′*-dimethylformamide and DMAc. The solubility of the polymer samples in various solvents was sufficient for the application of P2BO# and PABO as thin-film materials. Among the organic solvents, THF was chosen as the coating solvent for thin-film fabrication because of its low eco-toxicity and good biodegradability [69]. In addition, these polymer thin films can be fabricated at a low temperature using a wet process, owing to their good solubility in volatile organic solvents.

**Figure 3:**
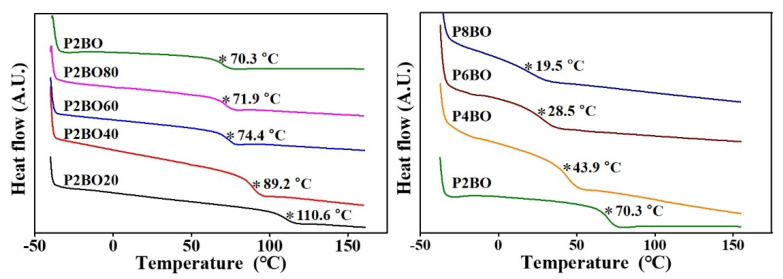
Differential scanning calorimetry (DSC) thermogram of P2BO# (P2BO20, P2BO40, P2BO60, and P2BO80) and PABO (P2BO, P4BO, P6BO, and P8BO) (* indicates the glass transition).

The thermal properties of the polymers were studied using DSC at a heating and cooling rate of 10 °C/min under a nitrogen atmosphere. All polymers were amorphous, and only one glass transition temperature was determined from the extrapolated intersection of the asymptotes for the glassy and rubbery regions for enthalpy (Figure 3) [70,71]. It has been demonstrated that the *T_g_* values of polymers can be increased by manipulating the polarity of the side group and that the values can be increased or decreased with an increase in side group bulkiness [72,73]. As the molar content of the *n*-alkylbenzoate-*p*-oxymethyl side group increased from 20 mol% to 100 mol%, the *T_g_* value decreased from 110.6 °C for P2BO20 to 70.3 °C for P2BO. In addition, as the number of carbon atoms in the alkyl moiety of the *n*-alkylbenzoate-*p*-oxymethyl side group increased from 2 to 8, the *T_g_* value decreased from 70.3 °C for P2BO to 19.5 °C for P8BO (Table 1). These results can be interpreted as an increase in the free volume of the polymers, which is influenced by the grafting of the *n*-alkylbenzoate-*p*-oxymethyl side group. Intermolecular and/or intersegmental interactions of the polymers are weaker with increasing distance between the polymer chain segments, which can lead to a decrease in *T_g_*.

**Figure 4:**
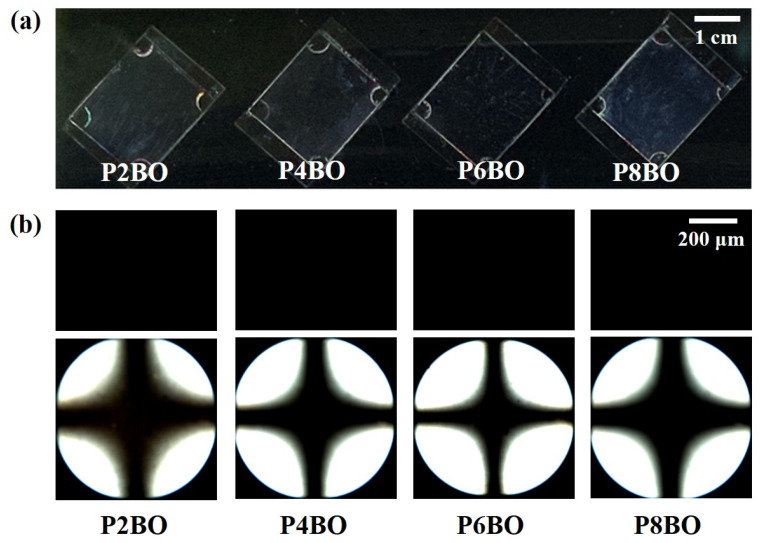
(**a**) Photograph, (**b**) orthoscopic (top) and conoscopic (bottom) polarized optical microscopy (POM) images of the LC cells fabricated with P2BO, P4BO, P6BO, and P8BO films.

Owing to the interactions between the LC molecules and the orientation layer, the molecular orientation of LC molecules can be induced by the chemical composition of the latter [74,75]. The polymer having even-number alkyl side chains can be preferrable as vertical orientation layer for liquid crystal than the orientation layer with odd-number methylene groups in the side chains [76,77]. Therefore, LC cells made from films of PS derivatives, substituted with precursors of LC molecules with an even number of methylene groups, such as ethylbenzoate-*p*-oxymethyl, *n*-butylbenzoate-*p*-oxymethyl, *n*-hexylbenzoate-*p*-oxymethyl, and *n*-octylbenzoate-*p*-oxymethyl in the side chain, were fabricated using 5CB in order to investigate the orientation behavior of the LC molecules on polymer films with LC-like moieties. Figure 4a shows a photograph of the LC cells fabricated with the PABO films, including P2BO, P4BO, P6BO, and P8BO. These LC cells showed uniform vertical LC orientation behavior over the entire area under two crossed polarizers. POM images of the LC cells were obtained in the orthoscopic (top) and conoscopic (bottom) modes (Figure 4b) to systematically investigate the orientation of the LC molecules. The LC cells were vertically oriented and exhibited a Maltese cross pattern in the conoscopic POM images. In addition, the LC cells assembled with the PABO films exhibited orientation stability over several months. The vertical orientation of the LC molecules may be influenced by the similarity of the molecular structures between the orientation layer and the LC molecules.

**Figure 5:**
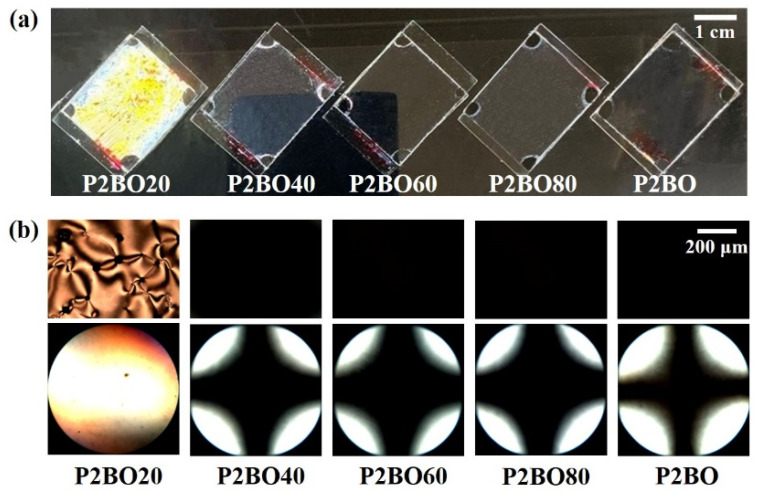
(**a**) Photograph, (**b**) orthoscopic (top) and conoscopic (bottom) polarized optical microscopy (POM) images of the LC cells fabricated with P2BO# (P2BO20, P2BO40, P2BO60, and P2BO80) and P2BO.

We also observed the orientation behavior of the LC molecules in P2BO# cells using 5CB to investigate the effect of the molar content of the ethylbenzoate-*p*-oxymethyl side group in the polymer. Figure 5a shows photographs of the LC cells made from P2BO# and P2BO films. The P2BO20 LC cells exhibited LC textures with birefringence, while uniform vertical LC orientation was observed for P2BO40, P2BO60, P2BO80, and P2BO over the entire area. Moreover, the LC orientation behavior of the LC cells fabricated with the P2BO# and P2BO films was investigated by observing their POM images for a more accurate analysis (Figure 5b). A random planar or a partial vertical LC orientation was observed in the LC cells fabricated using the P2BO20 film. However, P2BO# with 40 mol% or more of ethylbenzoate-*p*-oxymethyl side groups (P2BO40, P2BO60, and P2BO80) and P2BO films produced stable vertical LC orientation, as shown by the dark orthoscopic images and the Maltese cross patterns observed in the conoscopic images. The Maltese cross patterns in the conoscopic POM images of the P2BO40, P2BO60, P2BO80, and P2BO films indicate that there are no discernible differences in the LC orientation of polymers with different molar fractions of ethylbenzoate-*p*-oxymethyl in their side groups. The vertical orientation of the LC cells was observed for P2BO40, which contained only 40 mol% ethylbenzoate-*p*-oxymethyl groups. This result suggests that high similarity between the molecular structure of the LCs and the alignment layer assists with the vertical orientation of the LC molecules.

**Figure 6:**
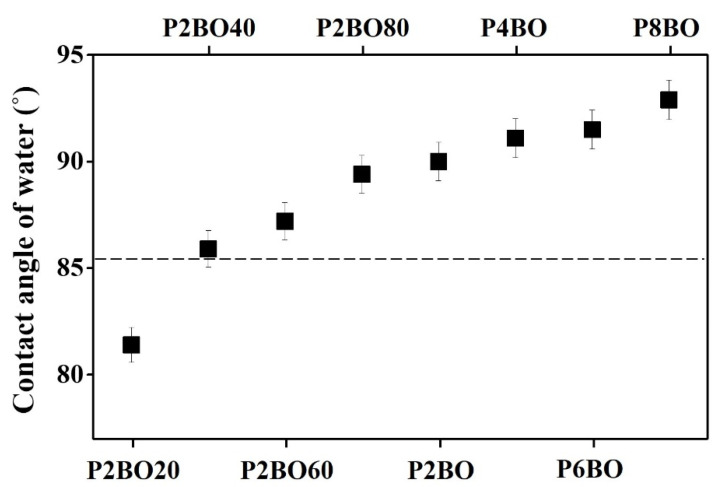
Water contact angle values and LC alignment behaviors of polymer films. Above and below the dashed line indicates vertical and planar LC orientation behaviors, respectively.

An understanding of the relationship between the orientation behavior of LC molecules and the surface properties of polymer films is important for the application of LCs in several fields. It is known that LC molecules can be oriented vertically on the substrate surface when the surface energy of the substrate is smaller than the surface tension of the LC [78,79,80]. It is well known that the low surface energy of polymer films, hydrophobicity of the surface, and low wettability can result in high contact angles for water on polymer films [81,82,83]. Therefore, the static contact angle of water on the P2BO# and PABO films was measured to investigate the effect of wettability on the LC orientation (Table 2 and Figure 6). Vertical orientation was observed for the LC molecules on P2BO40, P2BO60, P2BO80, P2BO, P4BO, P6BO, and P8BO films. The contact angles of water on the polymer films were 86°, 87°, 89°, 90°, 91°, 92°, and 93°, respectively. However, the LC cells fabricated with P2BO20, which had a water contact angle of 81°, did not reveal uniform vertical LC orientation behavior. Therefore, the vertical LC orientation behavior could be ascribed to the hydrophobic character of the polymer film, such as when the water contact angle is greater than approximately 86°.

**Figure 7:**
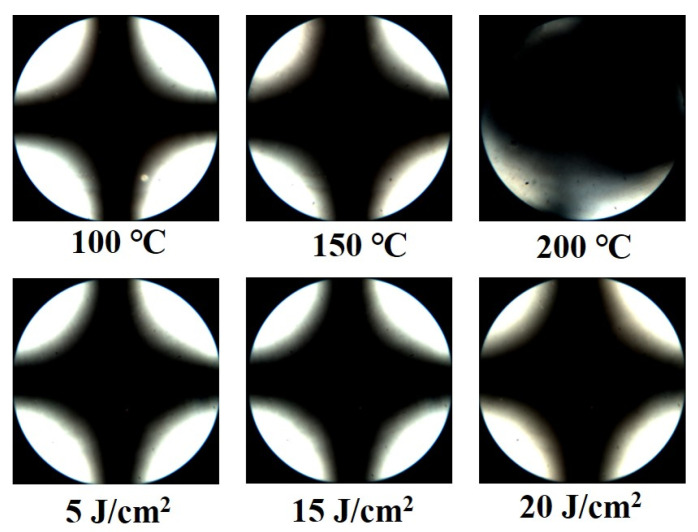
Conoscopic polarized optical microscopy (POM) images of the LC cells made using P2BO films after thermal treatment at 100, 150, and 200 °C for 10 min and UV treatment at 5, 15, and 20 J/cm^2^, respectively.

It was considered appropriate to use chemically inert homopolymers (PABO) to fabricate the LC devices because of the chemical reactivity of the chloromethyl group in the copolymer (P2BO#). Among the series of prepared polymers, P2BO was chosen as a promising material to orient the LC molecules, as it showed stable bulk thermal properties, confirmed by high *T_g_* values, and satisfactory hydrophobicity to allow the fabrication of LC cells by the capillary method. Therefore, the reliability evaluation of the LC cells fabricated with P2BO was conducted under harsh conditions, such as high temperatures and UV irradiation, in order to substantiate the LC orientation stability. Figure 7 shows the thermal stability behavior of the LC cells fabricated using P2BO films estimated from conoscopic POM images after heating for 10 min at temperatures of 100, 150, and 200 °C. The POM images of the LC cells fabricated with the polymer film indicate that the vertical LC orientation was maintained for 10 min at 150 °C, which is therefore the maximum processing temperature of this polymer for LC cell applications. In addition, the UV stabilities of the LC cells fabricated with the P2BO film were estimated from conoscopic POM images. Conoscopic POM images of the LC cells were captured after UV irradiation at 5, 10, and 20 J/cm^2^. As shown in Figure 7, no discernible differences in the vertical LC orientation on the P2BO film were observed in the conoscopic POM images, indicating that the vertical LC alignment of the LC cells was maintained even under high UV irradiation.

## 4. Conclusions

A copolymer series of ethylbenzoate-*p*-oxymethyl-substituted polystyrenes (P2BO# and homopolymer *n*-alkylbenzoate-*p*-oxymethyl-substituted polystyrenes (P2BO, P4BO, P6BO, and P8BO) were synthesized to evaluate the LC orientation behavior of polymer films. Vertical LC orientation behavior was observed for the LC cells made from polymers with a higher molar content of ethylbenzoate-*p*-oxymethyl side groups. For example, the LC cells fabricated with polymers with 40 mol% or more of ethylbenzoate-*p*-oxymethyl (P2BO40, P2BO60, P2BO80, and P2BO) side groups exhibited vertical LC orientation, while the LC cells fabricated from P2BO films with less than 20 mol% of ethylbenzoate-*p*-oxymethyl side groups revealed partial LC textures with birefringence. Vertical orientation was observed for the LC molecules in cells fabricated from polymer films, despite the short side chain length of P2BO and the low substitution ratio of approximately 40 mol%. These results suggest that similarity between the chemical structure of the orientation layer and LC molcules can be beneficial for achieving vertical orientation in LC molecules. The vertical LC orientation behavior correlated well, and the polymer films had a water contact angle larger than approximately 86°, owing to the unique structure of the *n*-alkylbenzoate-*p*-oxymethyl side chain. These polymer series can dissolve, readily, in medium-polarity solvents with low boiling points, such as tetrahydrofuran or chloroform. In addition, high temperature treatment for the baking process stage of the film fabrication is not demanded at all, as opposed to a conventional orientation layer made from polyimide derivatives. Therefore, we believe that the PABO polymer series are potential candidates for an LC orientation layer for next-generation applications with low temperatures based on wet processes.

## Figures and Tables

**Table 1:** Reaction conditions and results for the synthesis of the P2BO# and PABO (P2BO, P4BO, P6BO, and P8BO).

Polymer Designation	Feed Ratio of *n*-alkyl-*p*-hydroxybenzoate (mol%)	Degree of Substitution (mol%, ±10%)	*T*_g_ (°C)
P2BO20	20	20	110.6
P2BO40	40	40	89.2
P2BO60	60	60	74.4
P2BO80	80	80	71.9
P2BO	150	100	70.3
P4BO	150	100	43.9
P6BO	150	100	28.5
P8BO	150	100	19.5

**Table 2:** Water contact angle and LC alignment behavior of the polymer films.

Polymer Designation	Water Contact Angle (°) *^a^*	Vertical LC Aligning Ability *^b^*
P2BO20	81	X
P2BO40	86	O
P2BO60	87	O
P2BO80	89	O
P2BO	90	O
P4BO	91	O
P6BO	92	O
P8BO	93	O

*^a^* Measured from static contact angles. *^b^* O: Uniform vertical LC alignment, X: Planar LC alignment.

## Data Availability

The data presented in this study are available on request from the corresponding author.

## Supplementary Materials

The following are available online at https://www.mdpi.com/article/10.3390/polym13132058/s1. Preparation of *n*-alkylbenzoate-*p*-oxymethyl modified polystyrenes; Figures S1–S8: ^1^H nuclear magnetic resonance (NMR) spectra of PCMS, P2BO20, P2BO40, P2BO60, P2BO80, P4BO, P6BO, and P8BO; Figures S9–S17: ^13^C NMR spectra of PCMS, P2BO20, P2BO40, P2BO60, P2BO80, P2BO, P4BO, P6BO, and P8BO.s
