# Supplementary material for: Vertical Orientation of Liquid Crystal on Polystyrene Substituted with n-Alkylbenzoate-p-oxymethyl Pendant Group as a Liquid Crystal Precursor
Source: Polymers (Basel). 2021 Jun 23;13(13):2058. doi: 10.3390/polym13132058 (PMC8271889; doi:10.3390/polym13132058)
Supplement: Supplementary file 1 [file polymers-13-02058-s001.zip › polymers-1263959-supplementary.pdf]

Vertical orientation of liquid crystal on polystyrene substituted with *n*-alkylbenzoate-*p*-oxymethyl pendant group as a liquid crystal precursor

Kyutae Seo and Hyo Kang *

BK-21 Four Graduate Program, Department of Chemical Engineering, Dong-A University, 37 Nakdong-Daero 550 Beon-gil, Saha–gu, Busan 49315, Republic of Korea
* Correspondence: hkang@dau.ac.kr, Phone: +82 51 200 7720, Fax: +82 51 200 7728

Preparation of *n*-alkylbenzoate-*p*-oxymethyl modified polystyrenes

1. P2BO20

The following procedure was used to synthesize P2BO20. A mixture of PCMS (0.300 g, 1.97 mmol), ethyl-*p*-hydroxybenzoate (0.065 g, 0.39 mmol, 20 mol% compared with PCMS), and potassium carbonate (0.065 g, 0.47 mmol, 120 mol% compared with ethyl-*p*-hydroxybenzoate used substituent) in DMAc (50 mL) was heated to 70 °C and magnetically stirred at 200 rpm under a nitrogen atmosphere. The substitution reaction lasted for 24 h. Thereafter, the solution mixture was cooled to room temperature and poured into methanol to obtain a white precipitate. The precipitate was further purified by several reprecipitations from DMAc solution into methanol, and then a Soxhlet extractor was used to remove potassium carbonate and the remaining salts with boiling methanol. P2BO20 was obtained in > 80% yield after drying under vacuum overnight.

^{1}H NMR of P2BO20 (400 MHz, $CDCl_3$, δ/ppm): δ = 0.74–2.45 (–*CH*$_2$–*CH*–Ph–CH_2–Cl, –*CH*$_2$–*CH*–Ph–CH_2–O–, –COO–CH_2–*CH*$_3$, 9H), δ = 4.18–4.41 (–Ph–COO–*CH*$_2$–CH_3, 2H), δ = 4.42–4.76 (–Ph–*CH*$_2$–Cl, 2H), δ = 4.72–5.12 (–Ph–*CH*$_2$–O–Ph–, 2H), δ = 6.00–8.20 (–CH_2–CH–*PhH*–CH_2–Cl, –CH_2–CH–*PhH*–CH_2–O–*PhH*–COO–, 12H).

2. P2BO40

The following procedure was used to synthesize P2BO40. A mixture of PCMS (0.300 g, 1.97 mmol), ethyl-*p*-hydroxybenzoate (0.131 g, 0.79 mmol, 40 mol% compared with PCMS), and potassium carbonate (0.131 g, 0.95 mmol, 120 mol% compared with ethyl-*p*-hydroxybenzoate used substituent) in DMAc (50 mL) was heated to 70 °C and magnetically stirred at 200 rpm under a nitrogen atmosphere. The substitution reaction lasted for 24 h. Thereafter, the solution mixture was cooled to room temperature and poured into methanol to obtain a white precipitate. The precipitate was further purified by several reprecipitations from DMAc solution into methanol, and then a Soxhlet extractor was used to remove potassium carbonate and the remaining salts with boiling methanol. P2BO40 was obtained in > 80% yield after drying under vacuum overnight.

^{1}H NMR of P2BO40 (400 MHz, $CDCl_3$, δ/ppm): δ = 0.66–2.60 (–*CH*$_2$–*CH*–Ph–CH_2–Cl, –*CH*$_2$–*CH*–Ph–CH_2–O–, –COO–CH_2–*CH*$_3$, 9H), δ = 4.08–4.39 (–Ph–COO–*CH*$_2$–CH_3, 2H), δ = 4.62–4.73 (–Ph–*CH*$_2$–Cl, 2H), δ = 4.73–5.33 (–Ph–*CH*$_2$–O–Ph–, 2H), δ = 6.02–8.16 (–CH_2–CH–*PhH*–CH_2–Cl, –CH_2–CH–*PhH*–CH_2–O–*PhH*–COO–, 12H).

3. P2BO60

The following procedure was used to synthesize P2BO60. A mixture of PCMS (0.300 g, 1.97 mmol), ethyl-*p*-hydroxybenzoate (0.196 g, 1.18 mmol, 60 mol% compared with PCMS), and potassium carbonate (0.196 g, 1.42 mmol, 120 mol% compared with ethyl-*p*-hydroxybenzoate used substituent) in DMAc (50 mL) was heated to 70 °C and magnetically stirred at 200 rpm under a nitrogen atmosphere. The substitution reaction lasted for 24 h. Thereafter, the solution mixture was cooled to room temperature and poured into methanol to obtain a white precipitate. The precipitate was further purified by several reprecipitations from DMAc solution into methanol, and then a Soxhlet extractor was used to remove potassium carbonate and the remaining salts with boiling methanol. P2BO60 was obtained in > 80% yield after drying under vacuum overnight.

^{1}H NMR of P2BO60 (400 MHz, $CDCl_3$, δ/ppm): δ = 0.71–2.59 (–CH_2–*CH*–Ph–CH_2–Cl, –CH_2–*CH*–Ph–CH_2–O–, –COO–CH_2–CH_3, 9H), δ = 4.18–4.39 (–Ph–COO–CH_2–CH_3, 2H), δ = 4.41–4.61 (–Ph–CH_2–Cl, 2H), δ = 4.73–5.14 (–Ph–CH_2–O–Ph–, 2H), δ = 6.03–8.07 (–CH_2–CH–*PhH*–CH_2–Cl, –CH_2–CH–*PhH*–CH_2–O–*PhH*–COO–, 12H).

4. P2BO80

The following procedure was used to synthesize P2BO80. A mixture of PCMS (0.300 g, 1.97 mmol), ethyl-*p*-hydroxybenzoate (0.263 g, 1.58 mmol, 80 mol% compared with PCMS), and potassium carbonate (0.262 g, 1.90 mmol, 120 mol% compared with ethyl-*p*-hydroxybenzoate used substituent) in DMAc (50 mL) was heated to 70 °C and magnetically stirred at 200 rpm under a nitrogen atmosphere. The substitution reaction lasted for 24 h. Thereafter, the solution mixture was cooled to room temperature and poured into methanol to obtain a white precipitate. The precipitate was further purified by several reprecipitations from DMAc solution into methanol, and then a Soxhlet extractor was used to remove potassium carbonate and the remaining salts with boiling methanol. P2BO80 was obtained in > 80% yield after drying under vacuum overnight.

^{1}H NMR of P2BO80 (400 MHz, $CDCl_3$, δ/ppm): δ = 0.71–2.55 (–CH_2–*CH*–Ph–CH_2–Cl, –CH_2–*CH*–Ph–CH_2–O–, –COO–CH_2–CH_3, 9H), δ = 4.20–4.40 (–Ph–COO–CH_2–CH_3, 2H), δ = 4.42–4.64 (–Ph–CH_2–Cl, 2H), δ = 4.70–5.17 (–Ph–CH_2–O–Ph–, 2H), δ = 6.09–8.16 (–CH_2–CH–*PhH*–CH_2–Cl, –CH_2–CH–*PhH*–CH_2–O–*PhH*–COO–, 12H).

5. P4BO

The following procedure was used to synthesize P4BO. A mixture of PCMS (0.300 g, 1.97 mmol), *n*-butyl-*p*-hydroxybenzoate (0.575 g, 2.96 mmol, 150 mol% compared with PCMS), and potassium carbonate (0.491 g, 3.55 mmol, 120 mol% compared with *n*-butyl-*p*-hydroxybenzoate used substituent) in DMAc (50 mL) was heated to 70 °C and magnetically stirred at 200 rpm under a nitrogen atmosphere. The substitution reaction lasted for 24 h. Thereafter, the solution mixture was cooled to room temperature and poured into methanol to obtain a white precipitate. The precipitate was further purified by several reprecipitations from DMAc solution into methanol, and then a Soxhlet extractor was used to remove potassium carbonate and the remaining salts with boiling methanol. P4BO was obtained in > 80% yield after drying under vacuum overnight.

^{1}H NMR of P4BO (400 MHz, $CDCl_3$, δ/ppm): δ = 0.86–1.81 (–CH_2–*CH*–Ph–CH_2–, –COO–CH_2–$(CH_2)_2$–CH_3, 10H), δ = 4.17–4.39 (–Ph–COO–

CH_2–CH_2–, 2H), δ = 4.70–5.06 (–Ph–CH_2–O–Ph–, 2H), δ = 6.09–8.11 (–CH_2–CH–*PhH*–CH_2–O–*PhH*–COO–, 8H).

6. P6BO

The following procedure was used to synthesize P6BO. A mixture of PCMS (0.300 g, 1.97 mmol), *n*-hexyl-*p*-hydroxybenzoate (0.658 g, 2.96 mmol, 150 mol% compared with PCMS), and potassium carbonate (0.491 g, 3.55 mmol, 120 mol% compared with *n*-hexyl-*p*-hydroxybenzoate used substituent) in DMAc (50 mL) was heated to 70 °C and magnetically stirred at 200 rpm under a nitrogen atmosphere. The substitution reaction lasted for 24 h. Thereafter, the solution mixture was cooled to room temperature and poured into methanol to obtain a white precipitate. The precipitate was further purified by several reprecipitations from DMAc solution into methanol, and then a Soxhlet extractor was used to remove potassium carbonate and the remaining salts with boiling methanol. P6BO was obtained in > 80% yield after drying under vacuum overnight.

^{1}H NMR of P6BO (400 MHz, $CDCl_3$, δ/ppm): δ = 0.76–1.87 (–CH_2–*CH*–Ph–CH_2–, –COO–CH_2–$(CH_2)_4$–CH_3, 14H), δ = 4.12–4.34 (–Ph–COO–CH_2–CH_2–, 2H), δ = 4.67–5.06 (–Ph–CH_2–O–Ph–, 2H), δ = 5.99–8.14 (–CH_2–CH–*PhH*–CH_2–O–*PhH*–COO–, 8H).

7. P8BO

The following procedure was used to synthesize P8BO. A mixture of PCMS (0.300 g, 1.97 mmol), *n*-octyl-*p*-hydroxybenzoate (0.741 g, 2.96 mmol, 150 mol% compared with PCMS), and potassium carbonate (0.491 g, 3.55 mmol, 120 mol% compared with *n*-octyl-*p*-hydroxybenzoate used substituent) in DMAc (50 mL) was heated to 70 °C and magnetically stirred at 200 rpm under a nitrogen atmosphere. The substitution reaction lasted for 24 h. Thereafter, the solution mixture was cooled to room temperature and poured into methanol to obtain a white precipitate. The precipitate was further purified by several reprecipitations from DMAc solution into methanol, and then a Soxhlet extractor was used to remove potassium carbonate and the remaining salts with boiling methanol. P8BO was obtained in > 80% yield after drying under vacuum overnight.

^{1}H NMR of P8BO (400 MHz, $CDCl_3$, δ/ppm): δ = 0.77–1.78 (–CH_2–*CH*–Ph–CH_2–, –COO–CH_2–$(CH_2)_6$–CH_3, 18H), δ = 4.16–4.33 (–Ph–COO–CH_2–CH_2–, 2H), δ = 4.74–5.03 (–Ph–CH_2–O–Ph–, 2H), δ = 5.95–8.18 (–CH_2–CH–*PhH*–CH_2–O–*PhH*–COO–, 8H).

Figure captions

Figure S1. ^{1}H nuclear magnetic resonance (NMR) spectrum of PCMS.

Figure S2. ^{1}H nuclear magnetic resonance (NMR) spectrum of P2BO20.

Figure S3. ^{1}H nuclear magnetic resonance (NMR) spectrum of P2BO40.

Figure S4. ^{1}H nuclear magnetic resonance (NMR) spectrum of P2BO60.

Figure S5. ^{1}H nuclear magnetic resonance (NMR) spectrum of P2BO80.

Figure S6. ^{1}H nuclear magnetic resonance (NMR) spectrum of P4BO.

Figure S7. ^{1}H nuclear magnetic resonance (NMR) spectrum of P6BO.

Figure S8. ^{1}H nuclear magnetic resonance (NMR) spectrum of P8BO.

Figure S9. ^{13}C nuclear magnetic resonance (NMR) spectrum of PCMS.

Figure S10. ^{13}C nuclear magnetic resonance (NMR) spectrum of P2BO20.

Figure S11. ^{13}C nuclear magnetic resonance (NMR) spectrum of P2BO40.

Figure S12. ^{13}C nuclear magnetic resonance (NMR) spectrum of P2BO60.

Figure S13. ^{13}C nuclear magnetic resonance (NMR) spectrum of P2BO80.

Figure S14. ^{13}C nuclear magnetic resonance (NMR) spectrum of P2BO.

Figure S15. ^{13}C nuclear magnetic resonance (NMR) spectrum of P4BO.

Figure S16. ^{13}C nuclear magnetic resonance (NMR) spectrum of P6BO.

Figure S17. ^{13}C nuclear magnetic resonance (NMR) spectrum of P8BO.

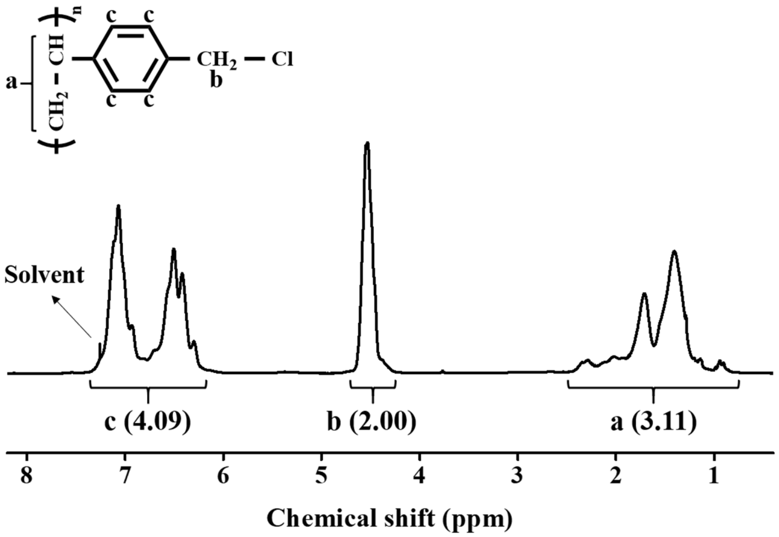

Figure S1. ¹H nuclear magnetic resonance (NMR) spectrum of PCMS.

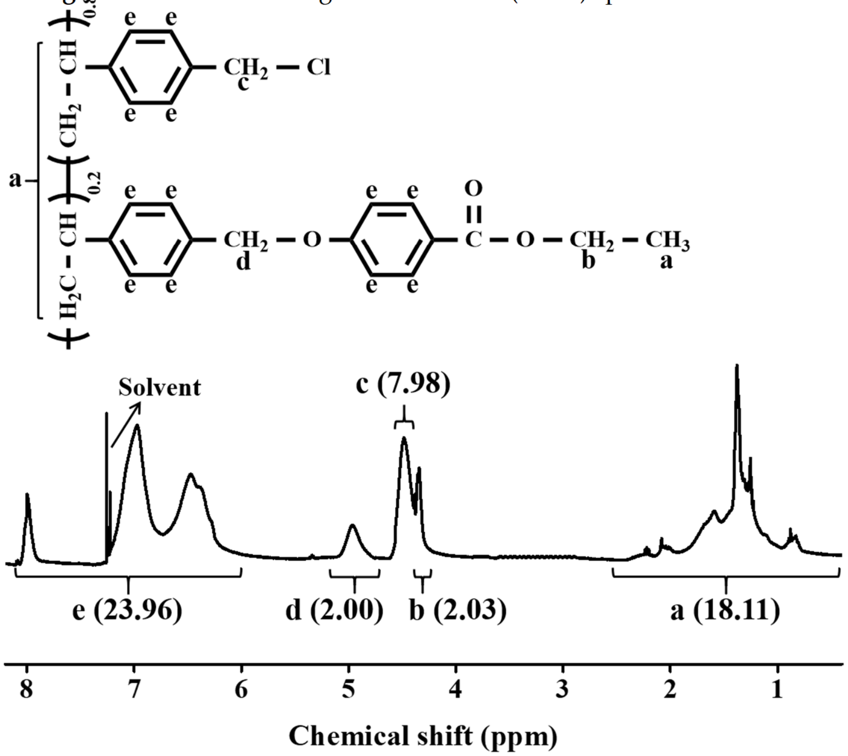

Figure S2. ¹H nuclear magnetic resonance (NMR) spectrum of P2BO20.

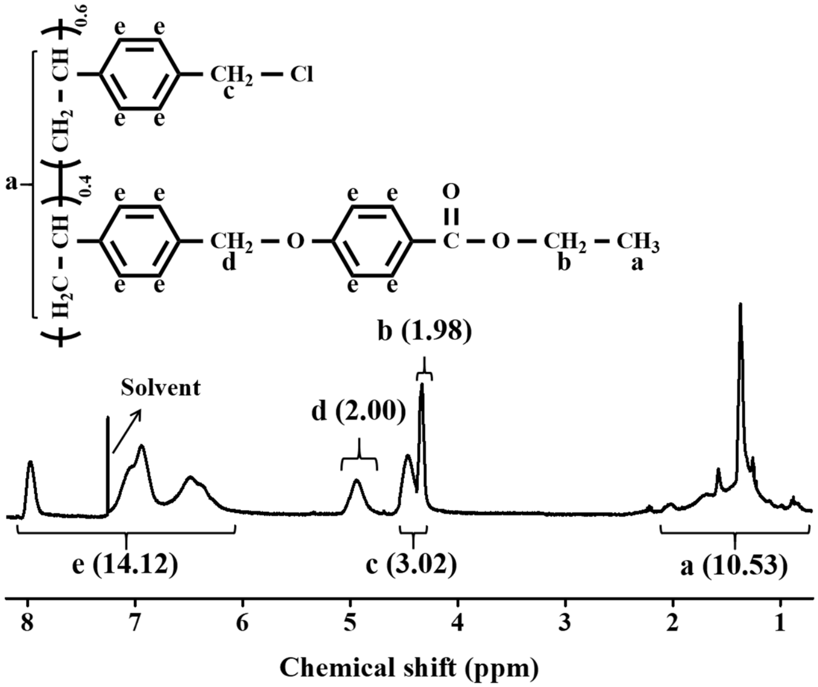

Figure S3. ¹H nuclear magnetic resonance (NMR) spectrum of P2BO40.

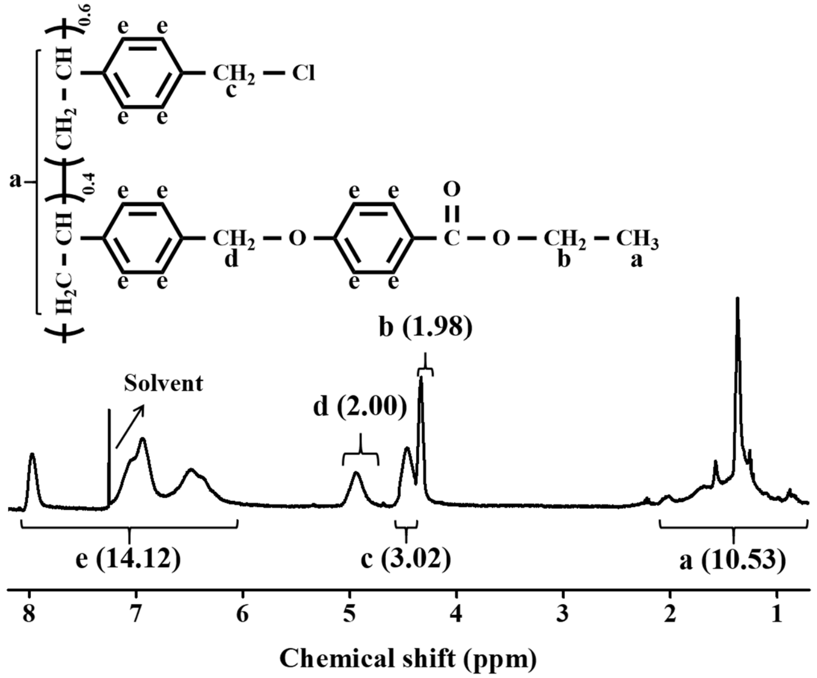

Figure S4. ^{1}H nuclear magnetic resonance (NMR) spectrum of P2BO60.

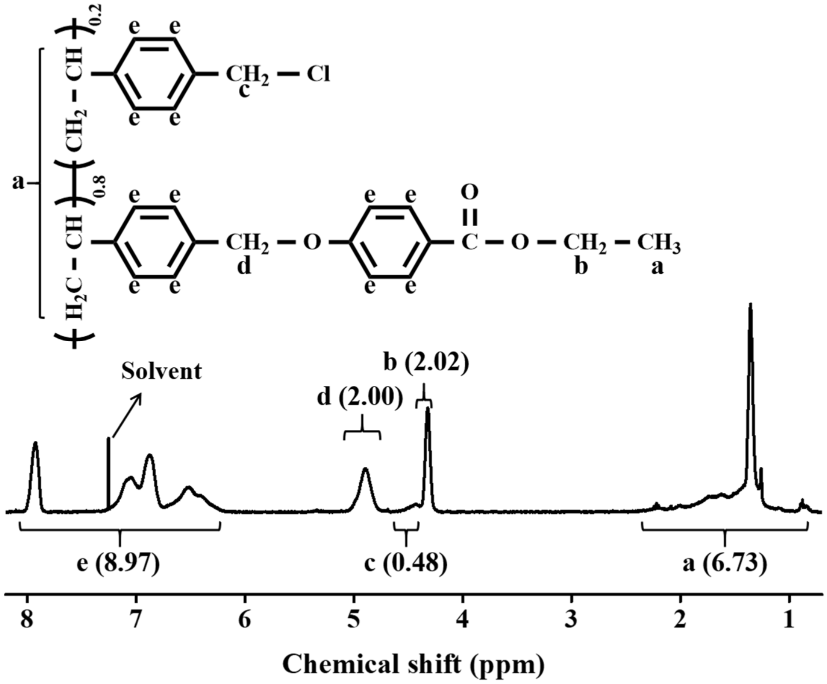

Figure S5. ^{1}H nuclear magnetic resonance (NMR) spectrum of P2BO80.

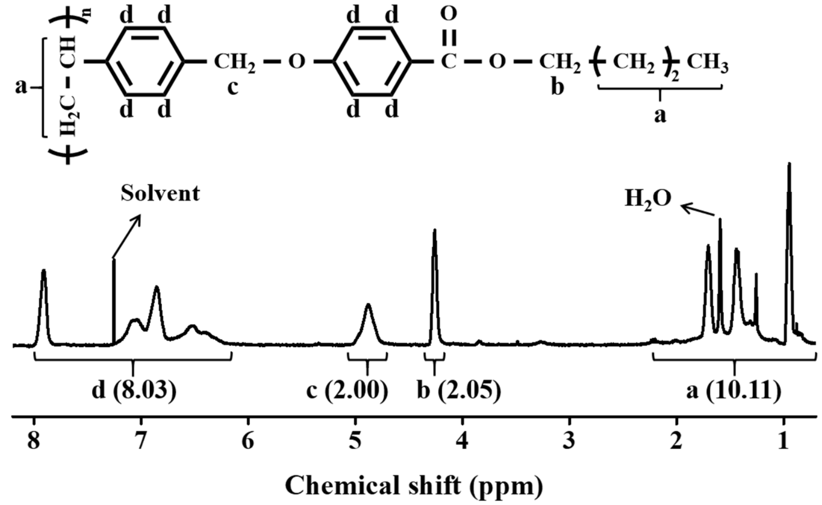

Figure S6. ^{1}H nuclear magnetic resonance (NMR) spectrum of P4BO.

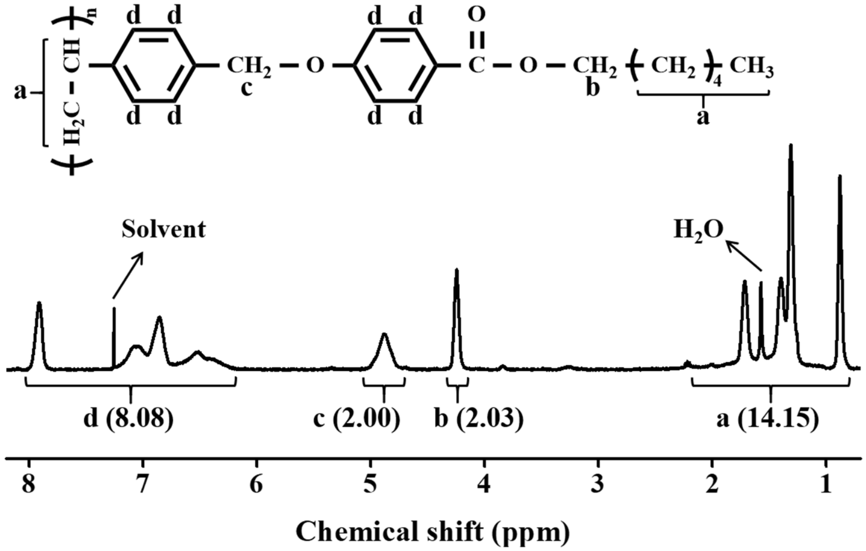

Figure S7. ^{1}H nuclear magnetic resonance (NMR) spectrum of P6BO.

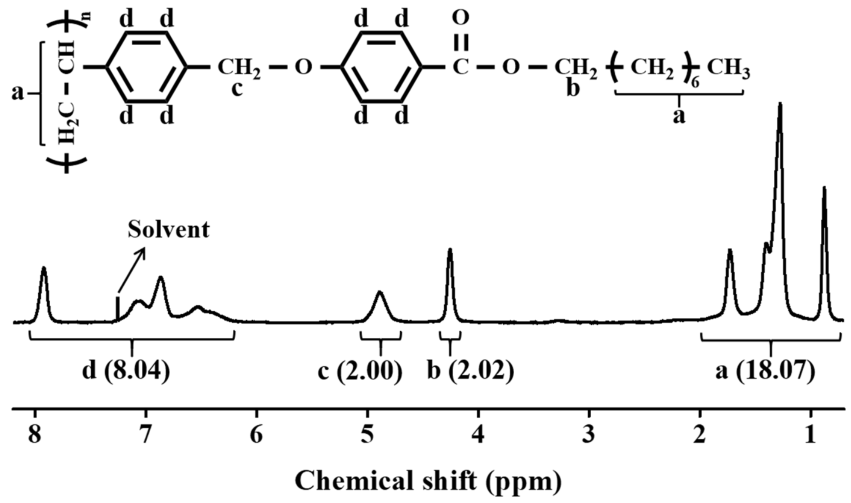

Figure S8. ^{1}H nuclear magnetic resonance (NMR) spectrum of P8BO.

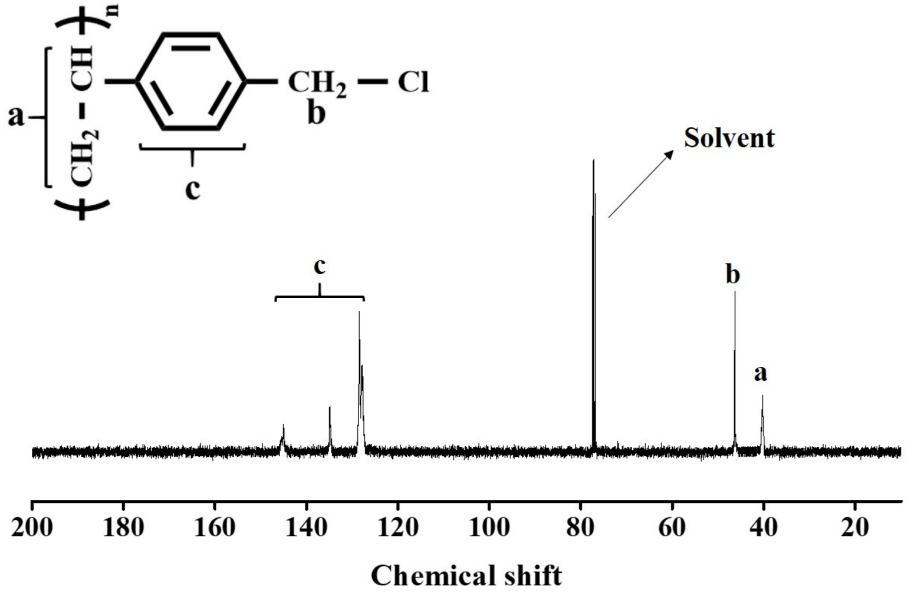

Figure S9. ^{13}C nuclear magnetic resonance (NMR) spectrum of PCMS.

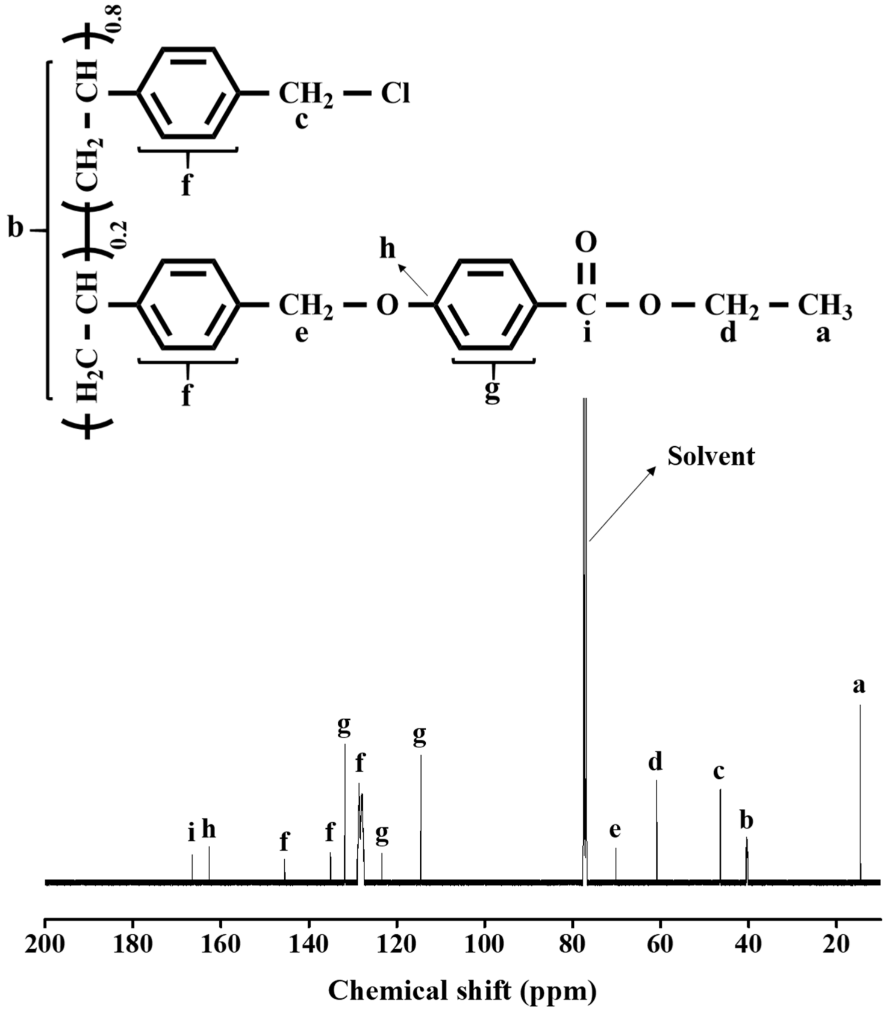

Figure S10. ^{13}C nuclear magnetic resonance (NMR) spectrum of P2BO20.

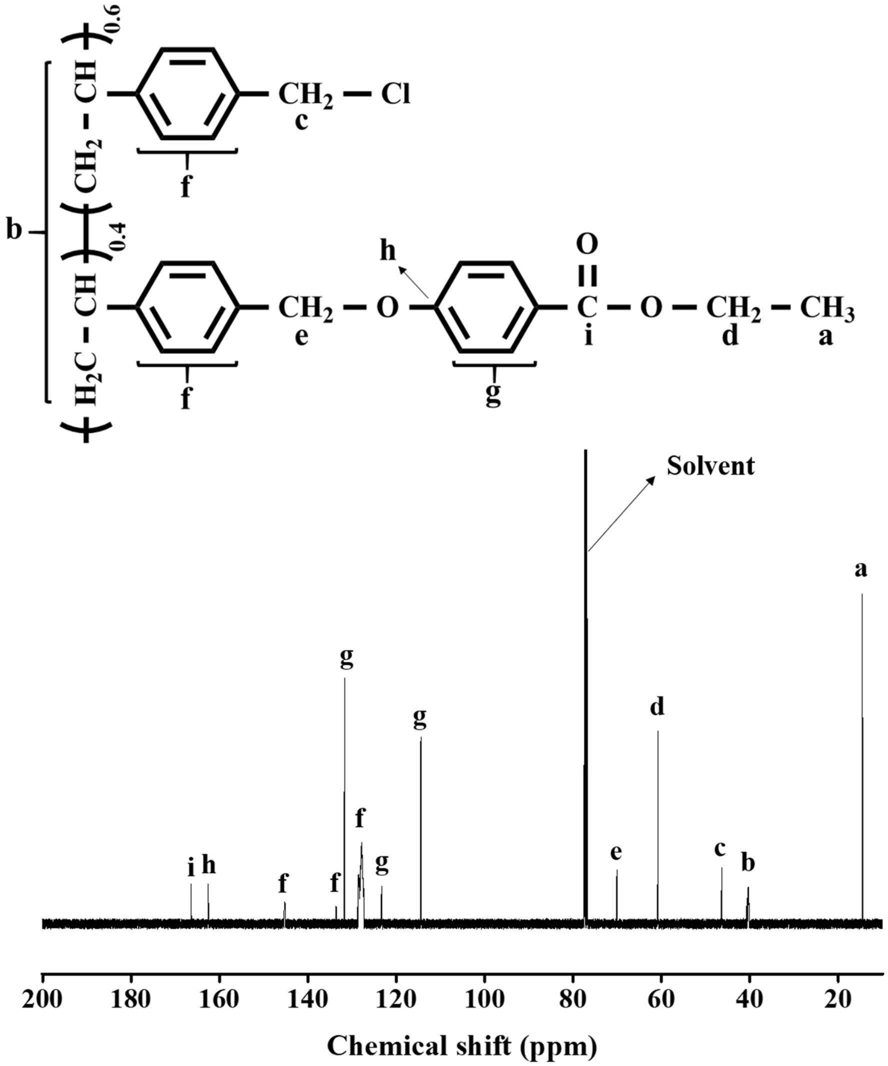

Figure S11. ^{13}C nuclear magnetic resonance (NMR) spectrum of P2BO40.

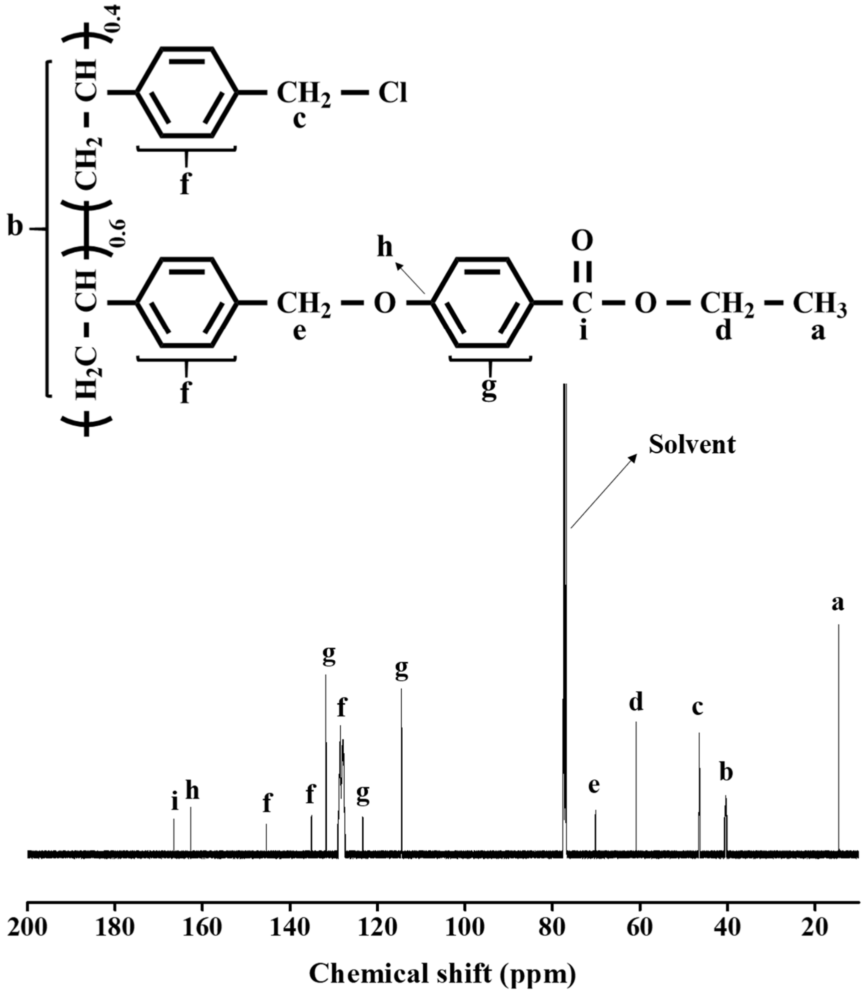

Figure S12. ^{13}C nuclear magnetic resonance (NMR) spectrum of P2BO60.

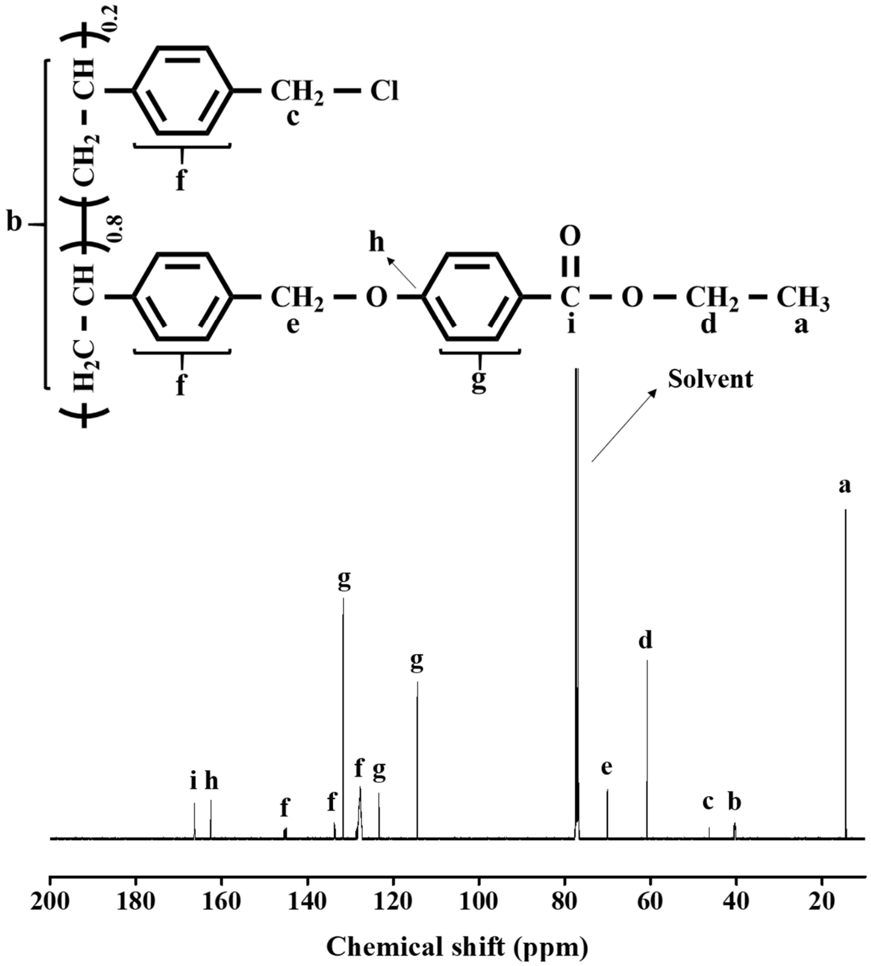

Figure S13. ^{13}C nuclear magnetic resonance (NMR) spectrum of P2BO80.

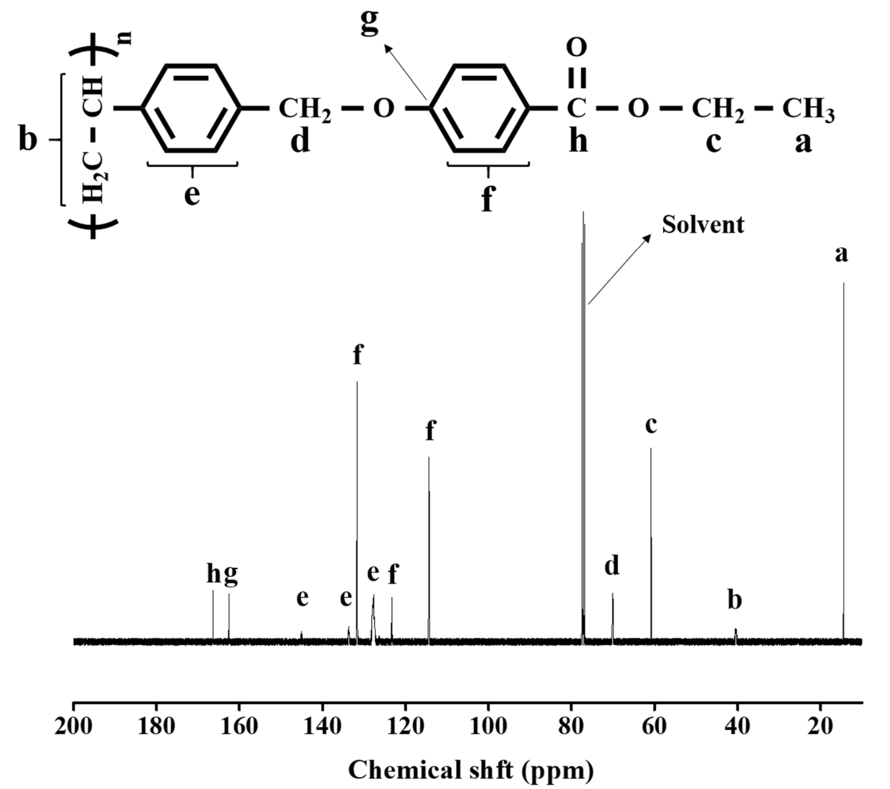

Figure S14. ^{13}C nuclear magnetic resonance (NMR) spectrum of P2BO.

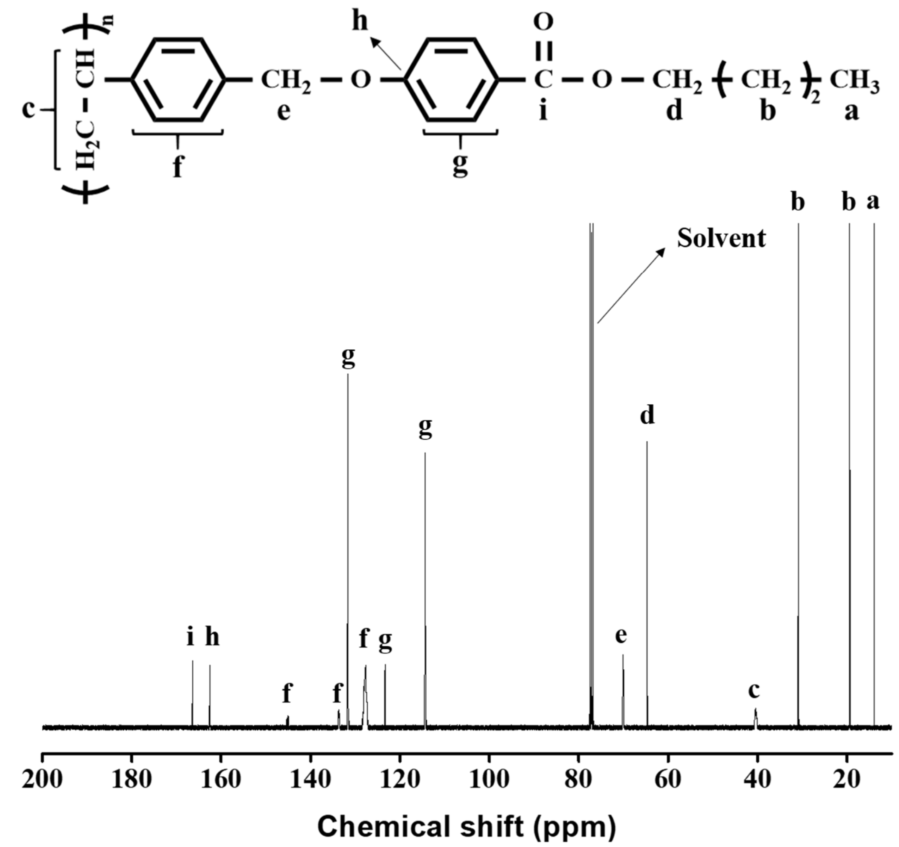

Figure S15. ^{13}C nuclear magnetic resonance (NMR) spectrum of P4BO.

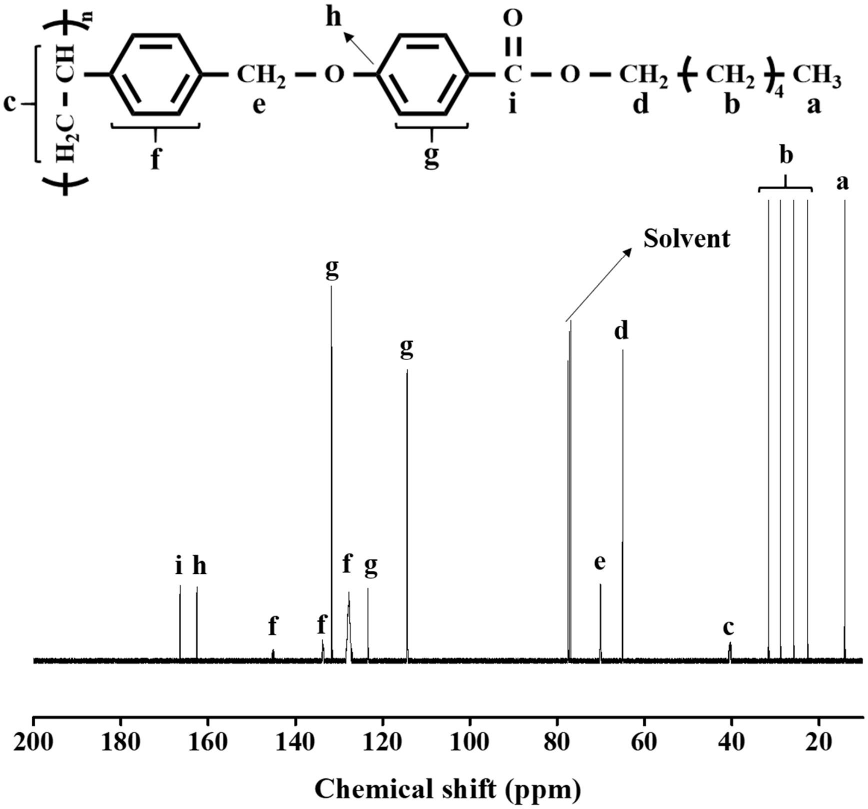

Figure S16. ^{13}C nuclear magnetic resonance (NMR) spectrum of P6BO.

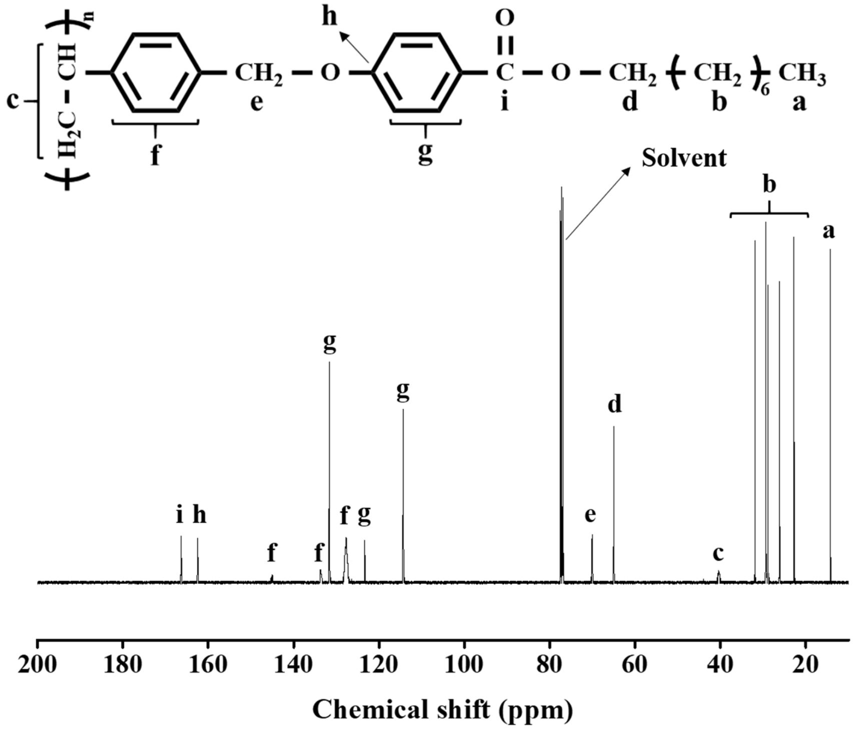

Figure S17. ^{13}C nuclear magnetic resonance (NMR) spectrum of P8BO.
